# Long‐term efficacy (at and beyond 1 year) of gastric peroral endoscopic myotomy for refractory gastroparesis: A systematic review and meta‐analysis

**DOI:** 10.1002/deo2.70021

**Published:** 2024-10-04

**Authors:** Francesco Vito Mandarino, Alberto Barchi, Noemi Salmeri, Francesco Azzolini, Ernesto Fasulo, Giuseppe Dell'Anna, Edoardo Vespa, Emanuele Sinagra, Jeremie Jacques, Silvio Danese

**Affiliations:** ^1^ Division of Gastroenterology and Gastrointestinal Endoscopy IRCCS San Raffaele Scientific Institute, Vita‐Salute San Raffaele University Milan Italy; ^2^ Gynecology/Obstetrics Unit, IRCCS San Raffaele Hospital Vita‐Salute San Raffaele University Milan Italy; ^3^ Gastroenterology and Endoscopy Unit, Fondazione Istituto G. Giglio, Contrada Pietra Pollastra Pisciotto Cefalu Italy; ^4^ Department of Gastroenterology and Endoscopy Dupuytren University Hospital Limoges France

**Keywords:** gastric peroral endoscopic myotomy, gastroparesis, G‐POEM, myotomy, refractory

## Abstract

**Introduction:**

Although gastric peroral endoscopic myotomy (G‐POEM) has shown substantial efficacy in patients with medically refractory gastroparesis (GP), comprehensive long‐term data on its effectiveness are lacking.

**Methods:**

We conducted a systematic review and meta‐analysis including observational studies assessing long‐term efficacy after G‐POEM in patients with refractory GP. Our primary outcome was the pooled rate of clinical success 1‐year after G‐POEM. Secondary outcomes included clinical success at 2 and 3 years and the rate of adverse events according to the American Society for Gastrointestinal Endoscopy classification.

**Results:**

Thirteen studies, involving 952 patients with refractory GP undergoing G‐POEM, were eligible. The pooled 1 year‐clinical success was 0.72 (95% confidence interval [CI]: 0.56, 0.85, I^2^ = 94.9%). The clinical success was 0.67 (95% CI: 0.47, 0.97, I^2^ = 95.8%) when considering only studies defining success as 1 point decrease in Gastroparesis Cardinal Symptoms Index score and at least 25% decrease in two subscales. For patients who had 1‐year success, the pooled clinical success at 2 and 3 years were 0.71 (95% CI: 0.45, 0.92, I^2^ = 94.9%) and 0.58 (95% CI: 0.19, 0.92, I^2^ = 97.1%), respectively. The pooled rate of adverse events was 0.08 (95% CI: 0.06, 0.10, I^2^ = 0%).

**Conclusion:**

G‐POEM is associated with successful outcomes in about 70% of treated cases after 1 year, with durable long‐term effects lasting up to 3 years. In the future, new uniform outcome definitions and strict patient selection criteria are warranted to delineate G‐POEM outcomes more accurately.

## INTRODUCTION

Gastroparesis (GP) is a chronic condition characterized by delayed gastric emptying (GE) of foods in the absence of mechanical obstruction of the stomach.^1^

Individuals with GP experience debilitating symptoms, such as vomiting, nausea, early satiety, and postprandial fullness, which can significantly decrease their quality of life. The healthcare costs associated with GP, including emergency room visits, inpatient admissions, and clinical consults, have dramatically increased in the last 20 years. In 2017, the estimated annual healthcare cost for GP in the United States was more than $500 million, and it is presumed to be on the rise.^2^

The etiology of GP is diverse and is associated with conditions like diabetes, post‐surgical states, autoimmune diseases, and neurological disorders. It is believed that viral infections are implicated in up to 20% of GP cases. However, in over 50% of cases, a single cause is not identified, leading to the classification of idiopathic GP.^3^

The growing interest in pyloric sphincter dysfunction as a mechanism of delayed GE in GP has prompted the development of pylorus‐targeted therapies for patients affected by disease refractory to pharmacological treatment. Over time, various endoscopic techniques have been developed. Although initially promising, botulinum toxin injection has not shown significant benefit compared to placebo,^4^ while pyloric dilation has resulted in symptom improvement in fewer than 50% of cases.^5^

Gastric peroral endoscopic myotomy (G‐POEM), introduced in 2013 by Khashab's group, has emerged as an effective treatment for refractory GP. G‐POEM potentially offers greater clinical success than surgical pyloromyotomy, with lower costs and shorter hospital stays.^6^

However, although the first randomized controlled trial on G‐POEM demonstrated the superiority over the sham procedure,^7^ there are still questions regarding the less‐than‐optimal success rate of the procedure. Additionally, evidence on long‐term outcomes and comprehensive data assessing the duration of G‐POEM's efficacy over time is lacking. This systematic review and meta‐analysis aims to evaluate the clinical efficacy of G‐POEM at 1 year, with a secondary focus on outcomes beyond 12 months.

## MATERIAL AND METHODS

### Protocol

We adhered to the Preferred Reporting Items for Systematic Reviews and Meta‐analysis (PRISMA) guidelines (Table S1) for this systematic review.^8^ The study protocol was prospectively registered at the International Prospective Register of Systematic Reviews (PROSPERO), accessible at https://www.crd.york.ac.uk/prospero (ID: CRD42022369842).

### Search strategy

We performed a systematic literature search from inception to May 2023 using PubMed, Medline, Embase, Web of Science, and Cochrane databases. The literature search was based on a combination of the following search terms: “*gastroparesis*”, “*myotomy*”, “*endoscopic treatment*”, and “*long‐term*”. The complete search strategy is detailed in Table S2.

We screened only English‐language and peer‐reviewed articles, removing duplicates using Rayyan (Clarivate), a bibliographic database manager. Article eligibility was initially assessed by titles and abstracts, followed by thorough full‐text reviews. The screening was conducted by Alberto Barchi and Emanuele Sinagra, and full‐text evaluations were by Francesco Vito Mandarino and Alberto Barchi Disagreements were reconciled by consulting a third investigator (Francesco Azzolini).

### Eligibility criteria

We included observational studies addressing outcomes of at least 1‐year post‐G‐POEM in patients with refractory GP. Exclusion criteria were pediatric cohorts, short‐term studies, non‐human studies, case reports, letters, narrative and systematic reviews, and studies lacking full text. Studies assessing efficacy beyond 1 year without data on 1‐year success were excluded.

### Data extraction

Data from original studies were extracted by two independent reviewers (Alberto Barchi and Ernesto Fasulo). Any discrepancies in data extraction were resolved under the supervision of Francesco Vito Mandarino.

The extracted data included: author; publication year; country; study design; patients’ number; demographic details (age, sex, and body mass index [BMI]); GP etiology; indication criteria for G‐POEM; prior endoscopic treatments; myotomy details; procedural time; technical success; clinical success definition; pre‐ and post‐operative Gastric Emptying Study (GES) and Gastroparesis Cardinal Symptom Index (GCSI) scores^9^; clinical success; adverse events (AEs); follow‐up duration. When available, pre‐ and post‐operative endoscopic functional luminal imaging probe (EndoFLIP) parameters and predictors of G‐POEM success or failure were also recorded.

### Quality assessment

The risk of bias in the studies was assessed using the Newcastle‐Ottawa Scale (NOS), a tool designed for assessing the quality of non‐randomized studies in meta‐analysis.^10^ The NOS score focuses on three key aspects, specifically the selection of study groups, the comparability of groups, and the ascertainment of the outcome. The risk of bias was evaluated by two independent reviewers (Francesco Vito Mandarino and Alberto Barchi), and a third reviewer (Emanuele Sinagra) in case of disagreement.

### Assessment of publication bias

Funnel plots inspection for asymmetry together with rank correlation tests (Begg's test) was used to assess publication bias in the analyses of 1‐year, and overall AEs. We decided to use the Begg test (or Rank Correlation Test) for several considerations: a) the limited number of studies included in this meta‐analysis and their observational design, which are known factors that can influence the results of the Egger test and increase the likelihood of false positives; and b) the Begg test is more suitable for small sample sizes, as it does not assume a normal distribution of standard errors.

Funnel plots and rank correlation tests were conducted using Jamovi (*Jamovi project*, version 2.3).

### Outcomes

Our primary outcome was the pooled rate of clinical success 1‐year after G‐POEM. Secondary outcomes included pooled rates of success at 2 and 3 years for patients who had clinical success at 1 year, the pooled mean GCSI score pre‐procedure, differences in GCSI score from baseline to post‐G‐POEM, pooled mean pre‐ and post‐procedural GES scores, differences in pre‐ and post‐procedural GES parameters, and the AEs rate.

The definition of clinical success varied slightly across studies. The most recent definition was a decrease of 1 point in the total GCSI score plus at least a >25% decrease in two subscales.^11^ However, due to the lack of a consensus, other definitions were also accepted and considered in subgroup analysis.

AEs were categorized following the American Society for Gastrointestinal Endoscopy (ASGE) lexicon classification.^12^

For studies on the same prospective cohort, pooled analysis of the secondary outcomes was obtained retaining only the larger cohort.

### Data synthesis and statistical analysis

For continuous outcomes, specifically GCSI scores pre‐, at 1‐ and 2‐years post‐procedure, and GES parameters pre‐ and post‐procedural, we calculated pooled mean difference (MD) with 95% CI.

Variables expressed as median and range or median and interquartile range (IQR) were converted into mean and standard deviation (SD) for pooling, using appropriate methods.^13^ After assessing skewness using the method by Shi et al.,^14^ if the data did not show significant skewness from normality, we extrapolated the mean from median values using the method by Luo et al.^15^ and calculated the SD from the IQR or range using the equation by Wan et al., when either of these measures was available.^16^ The equation by Shi et al. was applied if both the range and IQR were available.^14^ If the standard error of the mean (SEM) was available, SD was calculated using the Cochrane‐approved method: SD = SEM × √N.^17^

For binary outcomes, specifically clinical success at 1 year, 2 years, and 3 years, and AEs, we calculated pooled proportions with 95% confidence intervals (CIs). For 1‐year clinical success, the total number of patients with follow‐up was used as the denominator. For the analysis of post‐G‐POEM efficacy at 2 and 3 years, we used as denominator only the number of patients with clinical success at 1 year. When more than one study was conducted on the same clinical cohort, we included only the study with the largest available cohort.

Subgroup analyses for the primary outcome (clinical success at 1 year) were performed for studies enrolling patients with GP and Percent Gastric Retention (PGR) > 10% and for studies using the most recent definition of clinical success, as beforementioned.^11^ Leave‐One‐Out (LOO) sensitivity analyses were performed to evaluate the robustness of the overall effect measure.

The homogeneity of effect sizes among pooled studies was assessed using Cochran's Q test, with the corresponding p‐value reported alongside the I^2^ statistic. I^2^ values were interpreted according to Higgins et al. definition.^17^

The random effects model was applied in both the primary analyses and the subgroup analyses. This approach is indeed suitable for considering inter‐study variability in pooled estimates and is usually preferred in meta‐analysis of clinical studies where substantial heterogeneity is usually present.

Analyses were performed using the software Open Meta‐analyst (CEBM; Brown University).

Predictive factors of success and EndoFLIP parameters were also examined. Formal analyses of these data could not be performed as they were not consistently reported and were presented descriptively among the results.

## RESULTS

The literature search identified 1713 articles, from which 11 were removed as duplicates and 1618 were excluded after reviewing the title and abstract. Eighty‐four articles were then screened for inclusion criteria. Of these, 69 were excluded for various reasons: not being full articles (31 studies), having a follow‐up period shorter than 12 months post‐G‐POEM (20 studies), not reporting data on the primary outcome (15 studies), being non‐English (two studies), reporting data of 1‐year efficacy from the same cohort of patients (two studies), or reporting outcomes beyond 12 months without 1‐year efficacy data (one study). Ultimately, 13 studies comprising 952 patients with refractory GP undergoing G‐POEM were eligible for the systematic review and meta‐analysis^11^, ^18^, ^19^, ^20^, ^21^, ^22^, ^23^, ^24^, ^25^, ^26^, ^27^, ^28^, ^29^ (Figure S1). Tables 1, 2, 3, 4, 5 detail the baseline characteristics, endoscopic details, outcomes, and adverse events of the studies.

**TABLE 1:** Baseline characteristics.

									GP etiology, *n* (%)						Pre‐operative GES, mean (SD)		
Author	Year	Country	Study design	Multiple or single‐center	Patients (*n*)	Male, *n* (%)	Age, mean (SD)	BMI, mean (SD)	Diabetes	Idiopathic	Post‐surgery	Indication for G‐POEM	Previous endoscopic treatment, *n* (%)	Pre‐operative GCSI, mean (SD)	2 h PGR (%)	4 h PGR (%)	T ½ (min)
Abdelfatah et al	2021	USA	Retrospective	Single‐center	90	17 (19)	42.4 (12.6)	27.7 (7.4)	38 (42)	42 (47)	10 (11)	Refractory GP with 4 h PGR ≥ 10%	6 (6.7%)	3.8 (0.6)	NA	50.6 (27.3)	NA
Gregor et al.	2021	USA	Prospective	Single‐center	52	6 (11.5)	49.8 (15.9)	28.7 (7.2)	21 (40.4)	21 (40.4)	10 (19.2)	Refractory GP with 4 h PGR ≥ 10%	16 (43.2)	3.4 (1.0)	NA	36.5 (22.6)	NA
Hernandez Mondragon et al	2022	Mexico	Retrospective	Single‐center	374	141 (24.5)	48.4 (15.7)	26.4 (4.7)	141 (37.7)	115 (30.7)	102 (27.3)	Refractory GP with 4 h PGR > 10% and t ½ > 150 min	61 (10.6)	3.8 (0.5)	NA	40.8 (42.4)	246 med (150–368)
Hustak et al.	2020	Czech Republic	Retrospective	Single‐center	9	4 (44.4)	53.4 (37.6)	NA	3 (33.3)	1 (11.1)	5 (55.5)	Refractory GP with 2 h PGR ≥ 60%	NA	3.2 (0.8)	NA	NA	NA
Labonde et al.	2022	France	Retrospective	Multicenter	46	15 (32.6)	54 (15.9)	22.9 (5.4)	15 (32.6)	16 (34.8)	9 (19.6)	Refractory GP with 4 h PGR ≥ 10%	5 (10.9)	4.3 med (2.8–3.5)	NA	50.2 (27.6)	NA
Tan et al.	2021	China	Retrospective	Single‐center	79	57 (72.1)	65.7 (11.1)	19.7 (3.5)	0 (0)	0 (0)	79 (100)	Refractory GP with GCSI ≥ 2.3	NA	3.0 (0.6)	NA	NA	NA
Vosoughi et al.	2020	USA	Retrospective	Multicenter	37	13 (35.1)	56.2 (12.7)	25.9 (5.1)	12 (32.4)	10 (27)	10 (27)	Refractory GP	16 (43.2)	3.3 (1.1)	NA	NA	NA
Ragi et al.	2021	France	Retrospective	Multicenter	76	30 (39.5)	51.2 (37.7)	23.7 (6.0)	26 (34.2)	27 (35.5)	15 (19.7)	Refractory GP with h PGR 30% or 4 h PGR ≥ 10%	13 (17.1)	3.6 med (2.8–4.0)	79 (65–90)	45 (29–67)	180 med (140–280)
Vosoughi et al.	2022	USA	Retrospective	Multicenter	80	23 (28.7)	49.3 (14.9)	26.2 (6.0)	19 (23.8)	33 (41.3)	28 (35)	Refractory GP with impaired GE	56 (70)	2.8 (1.1)	NA	39 (22)	NA
Xu et al.	2018	China	Prospective	Single‐center	16	11 (68.7)	58.5 (16.1)	NA	3 (18.8)	0 (0)	13 (81.2)	Refractory GP with GCSI ≥ 20 and 2 h PGR ≥ 60%	0 (0)	NA	69.3 (11.5)	NA	183.2 (77.4)
Kahaleh et al.	2018	USA	Retrospective	Multicenter	33	11 (33.3)	52.5 (18.4)	NA	7 (21.2)	12 (36.4)	12 (36.4)	Refractory GP with abnormal GE	4 (12.1)	3.3	75.8	45	222.4
Reja et al.	2022	USA	Prospective	Single‐center	36	6 (16.7)	46.3 (1.75 SE)	NA	6 (16.7)	16 (44.4)	10 (27.8)	Refractory GP with delayed GE	NA	4.0 (0.12 SE)	NA	NA	275.1 (6.8 SE)
Conchillo et al.	2021	Netherlands	Prospective	Single‐center	24	3 (12.5)	55.3 (2.9)	NA	6 (25)	11 (45.8)	7 (29.2)	Refractory GP grade 3^*^	NA	3.1 (0.1)	NA	NA	240 (28)

Abbreviations: BMI, body mass index; GCSI, Gastric Cardinal Symptom Index; GE, gastric emptying; GES, gastric emptying study; GP, gastroparesis; med, median; NA, not available; PGR, percent gastric retention; SD, standard deviation; SE, standard error; t ½, half gastric time.

* nutritional status unable to be maintained via oral intake, and frequent hospital admissions.

**TABLE 2:** Endoscopic details and outcomes.

											Post‐operative GES, mean (SD)			
Author	MC location and myotomy type and	Procedural time, mean (SD) (min)	Technical success, *n* (%)	Definition of clinical success	1‐year clinical success, *n* (%)	2‐year clinical success, *n* (%)	3‐year clinical success, *n* (%)	GCSI at 12 months, mean (SD)	GCSI at 24 months, mean (SD)	GCSI at 36 months, mean (SD)	2 h PGR (%)	4 h PGR (%)	t ½ (min)	Follow‐up (months), mean (SD)
Abdelfatah et al.	MC at the greater curve. Single (circular muscle) or double layer myotomy (preserving outer longitudinal fibers) myotomy	50 (13)	90/90 (100)	↓ 1 point + 25% in 2 subscales in GCSI	44/83 (91.7)	20/21 (95.2)	6/7 (86)	2.3 (1.3)	1.6 (1.2)	1.1 (1.2)	NA	20.1 (23.5)	NA	36
Gregor et al.	MC at the greater curvature. Full‐thickness pyloromyotomy	59 (17)	52/52 (100)	↓ 1 point in GCSI	11/23 (48)	NA	NA	2.4 (1.6)	NA	NA	NA	10.2 (11.7)	NA	24
Mondragon et al.	MC at the greater or lesser curvature. Myotomy not detailed	55 (17.7)	374/374 (100)	↓ 1 point + 25% in 2 subscales in GCSI	323/331 (97.6)	293/303 (96.7)	203/214 (94.8)	1.6 (0.5)	1.6 (0.4)	1.9 (0.6)	NA	9 (1‐35)	112 (67–188)	48
Hustak et al.	MC at the greater curvature. Full‐thickness pyloromyotomy	75 (23)	9/9 (100)	↓ 40% in GCSI	8/9 (88.9)	3/4 (75)	NA	1.1 (0.6)	1.3 (0.8)	NA	NA	NA	NA	24
Labonde et al.	MC at the greater curvature. Full‐thickness pyloromyotomy	NA	46/46 (100)	↓ 1 point in GCSI	32/46 (69.5)	32/46 (69.5)	30/46 (65.2)	1.8	1.7	1.8	NA	NA	NA	36
Tan et al.	MC at the greater curvature. Full‐thickness pyloromyotomy	26.4 (4.2)	79/79 (100)	↓ 25% in 2 subscales in GCSI	47/60 (78.3)	27/33 (81.8)	NA	1.0 (0.5)	0.9 (0.4)	NA	NA	NA	NA	24
Vosoughi et al.	MC at the greater curvature. Selective myotomy of the pyloric circular muscle	50 med (41–65)	37/37 (100)	↓ 1 point + 25% in 2 subscales in GCSI	26/37 (70.3)	NA	NA	1.7 (1.2)	NA	NA	NA	NA	NA	12
Ragi et al.	MC at the greater curvature. Myotomy not detailed	47 med (28–78)	75/76 (98.6)	↓ 1 point in GCSI	50/76 (65.8)	29/39 (74.4)	NA	1.9 med (1–2.9)	NA	NA	52 (27)	11.5 (27)	100.5 (196)	24
Vosoughi et al.	MC at the greater curvature. Full‐thickness pyloromyotomy	43 med (34–56.5)	80/80 (100)	↓ 1 point + 25% in 2 subscales in GCSI	42/75 (56)	NA	NA	1.5 (1.2)	NA	NA		21 (27)	NA	12
Xu et al.	MC at the greater curvature. Full‐thickness pyloromyotomy	45.3 (12.3)	16/16 (100)	↓ 50% in GCSI	13/16 (81.3)	NA	NA	NA	NA	NA	33.4 (18.2)	NA	84.0 (34.7)	14 med (5–19)
Kahaleh et al.	MC at the greater curvature. Selective myotomy of the pyloric circular muscle	77.6 med (37‐255)	33/33 (100)	Improvement of GCSI	28/33 (85)	NA	NA	0.8 (0.8)	NA	NA	58.3	29.6	143.2	12
Reja et al.	MC at the greater curvature. Full‐thickness pyloromyotomy	60.5 med (35‐136)	36/36 (100)	Improvement of GCSI	31/36 (86)	NA	NA	1.94 (1.17 SE)	NA	NA	NA	NA	192.7 (1 SE)	15 (1.05 SE)
Conchillo et al.	NA	54 (5)	24/24 (100)	↓ 1 point in GCSI	8/24 (33.3)	NA	NA	2.4 (0.2)	NA	NA	NA	NA	228 (23)	12

↓decrease.

Abbreviations: GCSI Gastric Cardinal Symptom Index; GES, Gastric Emptying Study; med, median; NA, not available; SE, standard error.

**TABLE 3:** Adverse events according to the American Society for Gastrointestinal Endoscopy.

	Adverse events, *n* (%)			
Author	Mild*	Moderate*, *n* (%)	Severe*, *n* (%)	Description
Abdelfatah et al	2 (2.2)	2 (2.2)	0 (0)	Abdominal pain (one case), exacerbation of pre‐existing chronic respiratory disease (one case), capnoperitoneum (one case), and bleeding (one case)
Gregor et al.	3 (5.8)	0 (0)	0 (0)	Bleeding (one case) and leaks (two cases)
Hernandez Mondragon et al	26 (6.9)	7 (1.8)	0 (0)	Bleeding (17 cases), mucosal tear (eight cases), perforation (one case), clip dislodgment (two cases), and prepyloric ulcer (four cases)
Hustak et al.	0 (0)	2 (22.2)	0 (0)	Bleeding (one case) and leak (one case)
Labonde et al.	NA	NA	NA	NA
Tan et al.	7 (8.8)	0 (0)	0 (0)	Abdominal pain (seven cases)
Vosoughi et al.	5 (13.5)	0 (0)	0 (0)	Mucosotomy (two cases) and perforation (three cases)
Ragi et al.	7 (9.2)	0 (0)	1 (1.3)	Abdominal pain and fever (one case), persistent abdominal pain (one case), functional occlusion (two cases), perforation (one case), bleeding (two cases), and abdominal abscess (one case)
Vosoughi et al.	5 (6.2)	0 (0)	0 (0)	Abdominal pain (three cases), mucosotomy (one case), and thermal mucosa injury (one case)
Xu et al.	0	0 (0)	0 (0)	‐
Kahaleh et al.	2 (6)	0 (0)	0 (0)	Bleeding (one case) and ulcer (one case)
Reja et al.	5 (13.8)	0 (0)	0 (0)	Mucosotomy (five cases)
Conchillo et al.	2 (8.3)	1 (4.2)	0 (0)	Capnoperitoneum (two cases) and bleeding (one case)

* Based on the American Society for Gastrointestinal Endoscopy.

**TABLE 4:** Detailed adverse events.

	Adverse events, *n* (%)				
Author	Abdominal pain	Capnoperitoneum	Bleeding/ulcer	Mucosotomy/leak	Perforation
Abdelfatah et al.	1 (1.1)	1 (1.1)	1 (1.1)	0 (0)	0 (0)
Gregor et al.	0 (0)	0 (0)	1 (1.9)	2 (3.8)	0 (0)
Mondragon et al.	0 (0)	0 (0)	21 (5.6)	8 (2.1)	1 (0.3)
Hustak et al.	0 (0)	0 (0)	1 (11.1)	1 (11.1)	0 (0)
Labonde et al.	NA	NA	NA	NA	NA
Tan et al.	7 (8.8)	0 (0)	0 (0)	0 (0)	0 (0)
Vosoughi et al.	0 (0)	0 (0)	0 (0)	2 (5.4)	3 (8.1)
Ragi et al.	7 (9.2)	0 (0)	1 (1.3)	0 (0)	0 (0)
Vosoughi et al.	3 (3.8)	0 (0)	0 (0)	1 (1.3)	1 (1.3)
Xu et al.	0 (0)	0 (0)	0 (0)	0 (0)	0 (0)
Kahaleh et al.	0 (0)	0 (0)	2 (6.0)	0 (0)	0 (0)
Reja et al.	0 (0)	0 (0)	0 (0)	5 (13.9)	(0)
Conchillo et al.	0 (0)	2 (8.3)	1 (4.2)	0 (0)	0 (0)

**TABLE 5:** EndoFlip measures.

Author	Pre‐Endoflip DI 30 (mm^2^/mmHg), mean (SD)	Pre‐Endoflip DI 40 (mm^2^/mmHg), mean (SD)	Pre‐Endoflip DI 50 (mm^2^/mmHg), mean (SD)	Pre‐Endoflip CSA 30 (mm^2^), mean (SD)	Pre‐Endoflip CSA 40 (mm^2^), mean (SD)	Pre‐Endoflip CSA 50 (mm^2^), mean (SD)	Post G‐POEM Endoflip DI 30 (mm^2^/mmHg), mean (SD)	Post G‐POEM Endoflip DI 40 (mm^2^/mmHg), mean (SD)	Post G‐POEM Endoflip DI 50 (mm^2^/mmHg), mean (SD)	Post G‐POEM Endoflip CSA 30 (mm^2^), mean (SD)	Post G‐POEM Endoflip CSA 40 (mm^2^), mean (SD)	Post G‐POEM Endoflip CSA 50 (mm^2^), mean (SD)
Gregor et al.	7.2 (3.9)	6.5 (2.5)	4.7 (1.7)	83	122	171.6	9.1 (3.8)	9.7 (2.3)	6.9 (1.8)	97.7	162.2	226.1
Vosoughi et al.	NA	7.8 (4.9)	7.1 (3.7)	NA	119.1 (60.7)	176 (64.5)	NA	8.8 (6.1)	8.6 (6.1)	NA	146.5 (79)	206.9 (94.1)
Conchillo et al.	NA	5.3 med (3.1–8.1)	NA	NA	142.1 med (94.5–155.2)	NA	NA	7.5 (6.9–11.7)	NA	NA	159.2 (121.0–187.6)	NA

Abbreviations: CSA, cross‐sectional area; DI Distensibility Index; NA, not available.

### Quality assessment

Table S3 shows the methodologic quality of included studies according to NOS assessment. Specifically, 6 studies were considered of good quality, five of fair quality, and two of poor quality.

### Publication bias

The rank correlation tests for the 1‐year and overall AEs revealed no significant publication bias (*p* = 0.45 and 0.31, respectively). Funnel plots are shown in Figures S2 and S3.

### Clinical success at 1 year

Thirteen studies analyzed clinical success 1‐year post‐G‐POEM.^11^, ^18^, ^19^, ^20^, ^21^, ^23^, ^24^, ^25^, ^26^, ^27^, ^28^, ^29^ However, the study by Labonde et al.^23^ was not included in the analyses because it involved patients included in the cohort by Ragi et al.^25^

**FIGURE 1:**
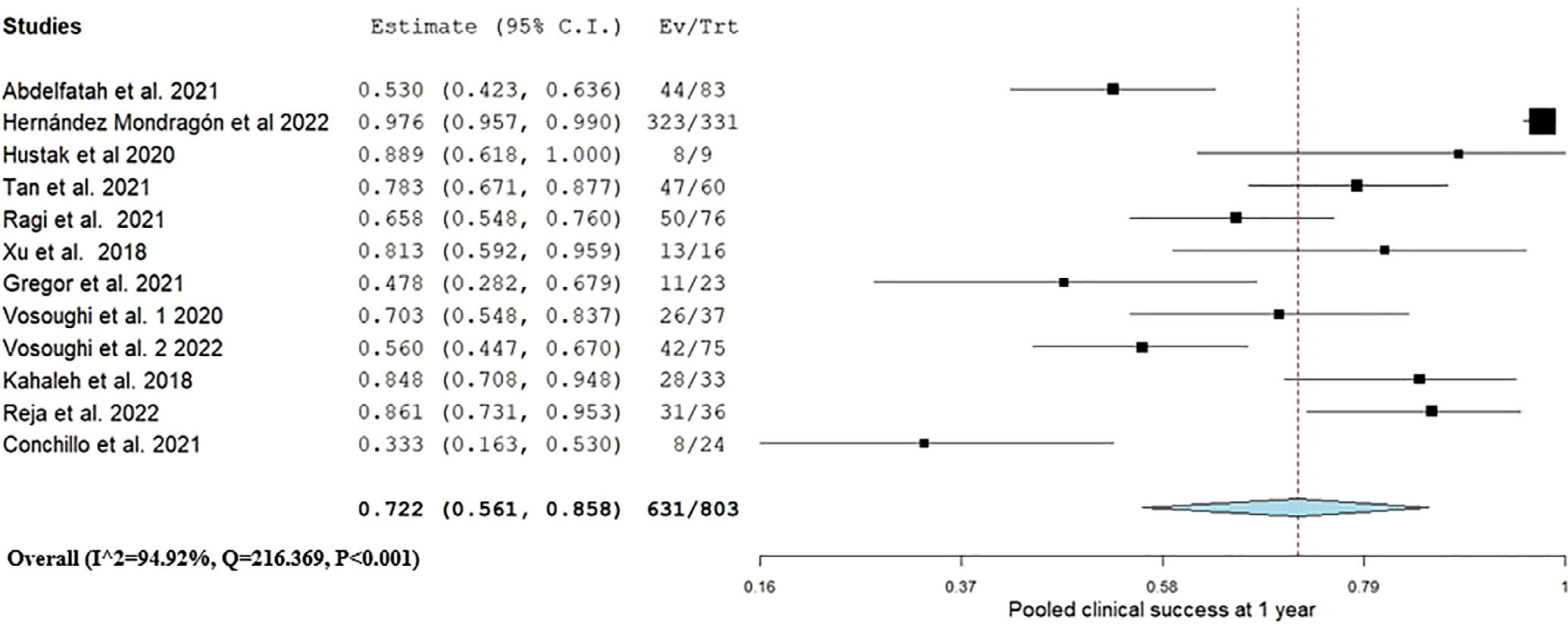
Pooled clinical success 1 year after gastric peroral endoscopic myotomy.

The pooled clinical success was 0.72 (95% CI: 0.56, 0.85, I^2^ = 94.9%) as shown in Figure 1. In LOO analyses, excluding the study by Tan et al.^25^ which only included patients with post‐surgical GP, the pooled 1‐year clinical success was 0.71 (95% CI: 0.55, 0.84; Figure S4).

In the subgroup analysis that included only studies defining GP as 4 h PGR ≥ 10%, the pooled clinical success was 0.61 (95% CI: 0.52, 0.70, I^2^ = 46.6%; Table S4).

In the subgroup analysis that included only studies defining success as a 1‐point decrease of total GCSI score and at least 25% decrease of two GCSI subscales‐ 4 studies,^11^, ^18^, ^20^, ^24^ the pooled clinical success was 0.67 (95% CI: 0.47, 0.97, I^2^ = 95.8%; Table S4).

### Clinical success at 2 and 3 years

**FIGURE 2:**
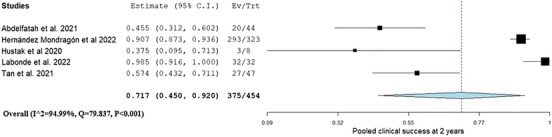
Pooled clinical success 2 years after gastric peroral endoscopic myotomy for patients who experienced success after the first year.

**FIGURE 3:**
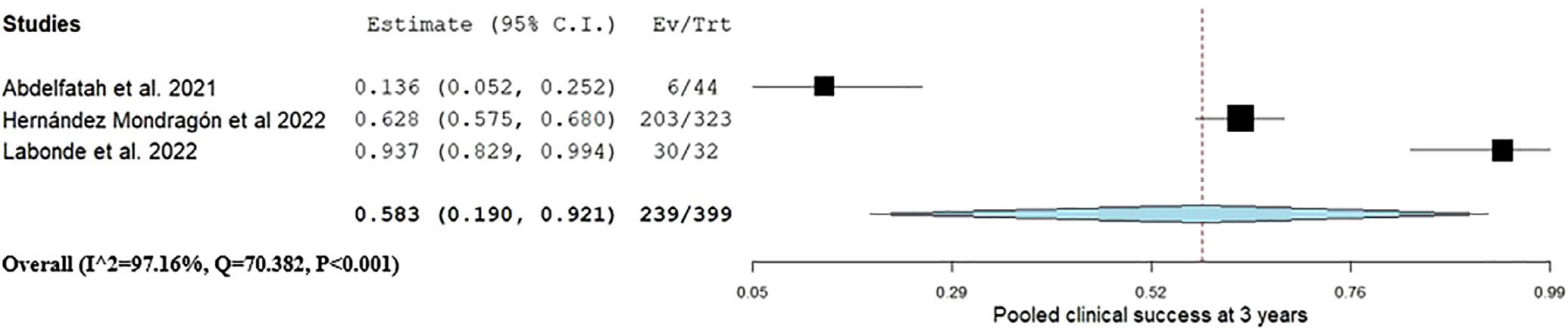
Pooled clinical success 2 years after gastric peroral endoscopic myotomy for patients who experienced success after the first year.

Clinical success 2 years post‐G‐POEM was reported in five studies.^18^, ^20^, ^21^, ^22^, ^23^ The pooled clinical success was 0.71 (95% CI: 0.45, 0.92, I^2^ = 94.9%) as shown in Figure 2. Clinical success at 3 years was assessed in three studies with reported 1‐year outcomes.^18^, ^20^, ^22^ The pooled clinical success was 0.58 (95% CI: 0.19, 0.92, I^2^ = 97.1%; Figure 3).

### GCSI score changes

The pooled mean GCSI scores before the procedure, 1‐year post‐G‐POEM, and 2 years post‐G‐POEM are shown in Table S4. The MD between the 1‐year post‐procedure and pre‐operative GCSI was −1.61 (95% CI: −2.18, −1.04, I^2^ = 98.6%). The MD between 2 years’ post‐procedure and pre‐operative GCSI was −2.18 (95% CI: −2.25, −2.12, I^2^ = 0%; Table S4).

### GES parameters

The pooled mean gastric half‐emptying time (t ½) and 4‐h PGR scores before and after the procedure are presented in Table S4. The pooled MD in gastric half time between pre‐operative and post‐operative assessment was −106.82 min (95% CI: −183.285, −30.363.73, 8.48, I^2^ = 98.8%). The pooled MD in 4‐h PGR between pre‐operative and post‐operative assessment was −27.3 (95% CI: −34.5, −20.0, I^2^ = 68.1%; Table S4).

### Adverse events

**FIGURE 4:**
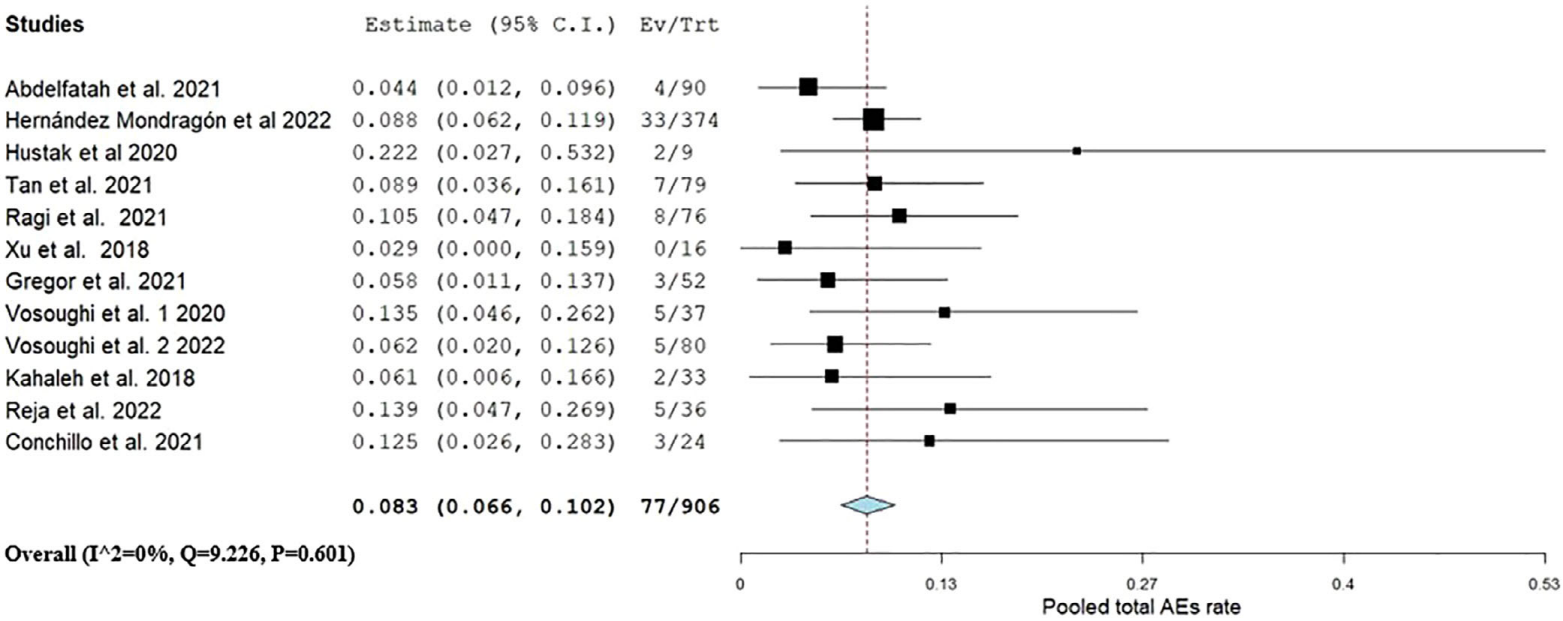
Pooled rate of overall adverse events.

Data on AEs were provided by 12 studies.^11^, ^18^, ^19^, ^20^, ^21^, ^23^, ^24^, ^25^, ^26^, ^27^, ^28^, ^29^ The pooled rate of AEs was 0.08 (95% CI: 0.06, 0.10, I^2^ = 0%) as shown in Figure 4). AEs according to the American Society for Gastrointestinal Endoscopy (ASGE) and detailed events are presented in Tables 3 and 4. The pooled rates for mild, moderate, and severe AEs were 0.06 (95% CI: 0.05, 0.08, I^2^ = 0%), 0.01 (95% CI: 0.00, 0.02, I^2^ = 0%), and 0.00 (95% CI: 0.00, 0.01 I^2^ = 0%), as presented in Table S4.

### Endoflip

Three studies provided EndoFLIP measurements in both pre‐ and post‐G‐POEM procedures.^19^, ^24^, ^29^ In all three studies, an increase in the Distensibility Index (DI) post‐G‐POEM was observed. Additionally, the study by Gregor et al. reported an increase in the post‐myotomy cross‐sectional area (CSA).^19^ Further details are provided in Table 5.

### Factors of predictive success and failure

In the study by Hernandez Mondragon et al., predictors of success included diabetic etiology, early diagnosis within 24 months, predominant symptoms of nausea and vomiting, and GCSI scores between 1.5 and 2.5 at 6 months.^20^ Vosoughi et al. found that CSA at 40‐mL (>154 mm^2^) and 50‐mL (>247.5 mm^2^) distention volumes at post‐G‐POEM EndoFLIP were associated with clinical success.^24^ Ragi et al. reported that high pre‐operative GCSI satiety subscale scores were predictors of success.^25^ Conversely, Abdelfatah et al. found that a higher BMI, the use of psychiatric and pain medications, a longer duration of GP, and higher pre‐operative nausea were associated with unfavorable outcomes (Table S5).^18^

## DISCUSSION

This meta‐analysis shows that the 1‐year clinical success of G‐POEM across 13 studies was equal to 72% (I^2^ = 94.9%). Consistent results were found when adopting only the most recent definition of success,^11^ with pooled 1‐year clinical success equal to 67.7% (I^2 =^ 95.85%). The substantial heterogeneity reflects the variability among the studies included in the meta‐analysis and arises from differences in study design, population characteristics, and success definition. To address this issue, we conducted subgroup analyses, which partially reduced the level of heterogeneity. When considering only studies enrolling patients with 4‐h PGR > 10%, the clinical success was slightly lower and equal to 61.0%, with I^2^ = 46.6%). The reduction of clinical success rate and heterogeneity may be interpreted as being due to a more accurate selection of patients with GP.

Our 1‐year clinical success after G‐POEM is similar to the short‐term success reported in the randomized controlled trial by Martinek and colleagues and in the previous meta‐analyses by Mohan et al., ranging from 71% to 75.8%.^7^, ^30^ Remarkably, it is higher than the 1‐year pooled clinical success of 61% reported in the meta‐analysis by Kamal et al., published in 2021.^31^ This might be due to evolving methodologies and increasing knowledge on GP, potentially leading to improved pooled success after G‐POEM.

According to our pooled estimates, G‐POEM maintained clinical efficacy for 71% of patients at 2 years and 58% at 3 years, who had achieved 1‐year clinical success. Our data suggest that G‐POEM may have a lasting clinical efficacy even beyond 1 year, albeit gradually diminishing over time.

Comprehensive long‐term data on G‐POEM are scarce. Only a recent meta‐analysis conducted by Canakis et al. analyzed the outcomes of G‐POEM beyond 3 years, reporting a clinical success of 75%. However, in the pooled analysis, the authors utilized the number of followed‐up patients from the initial cohort as the denominator, not considering patients with no follow‐up as clinical failures across all included studies. This approach could have potentially influenced the results.^32^

One of the critical issues with G‐POEM is identifying patients with refractory GP who could benefit from the procedure. In our systematic review, we analyzed predictive factors for the success of myotomy reported in long‐term studies. Factors such as GCSI >2.6 and clinical success at 1 month have been reported as predictors of clinical success at 12 months.^11^ High BMI, long duration of GP, and psychiatric or narcotic medication use have been associated with poor outcomes.^18^ However, at present, the outcome predictors of myotomy appear to be inconsistent and somewhat contradictory. For example, factors such as predominant nausea or a high 4‐h PGR have been identified as both a success factor,^11^, ^20^ and a failure factor.^22^, ^25^ These conflicting results make it challenging to establish clear guidelines for predicting outcomes. Currently, the evidence remains weak, and unfortunately, the implications for clinical practice are merely suggestive at this time. This highlights the need for further research to better define the predictors of success for G‐POEM and to standardize criteria for patient selection.

In recent years, new advancements like GES regional analysis and EndoFLIP, have emerged as techniques to identify GP patients who could benefit from pyloromyotomy. In a prospective study conducted by our group, lower median pre‐procedural Intragastric Meal Distribution at time 0 (indicating impaired fundic accommodation) was found to correlate with a higher G‐POEM success rate.^33^ Endoflip is a promising tool for identifying appropriate GP patients for G‐POEM in the realm of precision endoscopy.^34^ In the study conducted by Jacques et al., a preoperative EndoFLIP pylorus DI threshold of 9.2 mm^2^/mmHg was found to predict G‐POEM success with 72% sensitivity and 100% specificity.^35^ In our systematic review all studies analyzing the use of EndoFLIP showed an improved pyloric DI and an increase in CSA after G‐POEM. This suggests that the myotomy effectively alters pyloric function. However, this does not incontrovertibly correlate with symptom improvement. GP is a complex disease with multiple pathophysiological mechanisms contributing to GE impairment, which include, in addition to pylorospasm, impaired gastric accommodation, and antro‐duodenal dysmotility.^36^ The impact of these mechanisms on symptoms, still partially unknown, can vary, and it is plausible that not all may be addressed with pyloromyotomy. Further research is needed to better define the association between the organic change of the pyloric muscle after G‐POEM and symptom relief, as well as the role of EndoFLIP in myotomy patient selection.

The definition of post‐G‐POEM clinical success poses an additional challenge. Primarily, success following G‐POEM is evaluated using the GCSI score. However, this score might not be entirely suitable as it includes the assessment of non‐specific gastrointestinal symptoms that are also present in other functional diseases. Moreover, the arbitrary nature of the most recent definition of success^11^ raises questions about its clinical significance and validity. Nevertheless, it is important to note that the improvement of symptomatology remains the primary G‐POEM outcome, especially considering that GES has not been shown to correlate with GP symptoms.^37^ In this regard, our meta‐analysis shows long‐term improvements in the GCSI following G‐POEM, with an MD of −1.61 at 1 year and −2.18 at 2 years compared to the pre‐treatment score. Additionally, we found significant post‐operative improvements in GES parameters compared to the pre‐treatment (MD of t ½ −97.62 min and a 4‐h PGR reduction of −27.32). Collectively, our findings show that G‐POEM leads to clinical and functional improvements.

In our meta‐analysis, the pooled rate of AEs was 8.4% (6.9% mild and 1.5% moderate, according to the ASGE classification).^12^ This rate is similar to the 8% reported in the meta‐analysis by Kamal et al.^31^ Predominantly, AEs included bleeding/post‐myotomy ulcers (28 cases) and mucosotomy/leak (19 cases). Our data confirm that G‐POEM is a safe procedure, with very rare occurrences of severe AEs.

Our meta‐analysis is not without limitations. The pooled estimates in our analysis carry a moderate to high heterogeneity. As mentioned earlier, these mostly arise from methodological issues in existing long‐term studies on G‐POEM. Long‐term G‐POEM studies exhibit inconsistencies in defining clinical success and have varying follow‐up durations, which present significant challenges for conducting pooled analyses. However, to minimize this limitation, we performed a subgroup analysis including only studies that adopted the most recent definition of clinical success.^11^

In addition to the high heterogeneity, the relatively wide range of 95% CI is also a limitation of this study. The wide CIs reflect greater uncertainty about the estimated effect size, likely due to variability between studies and small sample sizes, especially in some of the included studies. This suggests that the true effect may vary, making it harder to draw definitive conclusions about the effect size. Therefore, caution should be exercised in interpreting the results.

Another concern is that in some studies not all patients from the initial cohort were followed up at 2 and 3 years. However, in calculating the pooled clinical success at 2–3 years, we have considered only the number of patients with clinical success at 12 months as the denominator. In our view, this analysis represents the most reliable method for estimating success at 2 and 3 years based on current studies.

Despite the limitations of the included studies, our meta‐analysis represents the most comprehensive summary of 1‐year clinical success of G‐POEM in nearly 1000 patients. It also presents pooled success data beyond 1 year in over 600 patients. Our results demonstrate that G‐POEM achieves clinical success at 1 year in approximately 70% of patients, which may be sustained but could decline over time. As we await new studies to clarify the pathogenesis of GP, there is an urgent need for standardized measures to facilitate the interpretation of G‐POEM outcomes.

## CONFLICT OF INTEREST STATEMENT

None.

## ETHICS STATEMENT

- **Approval of the research protocol by an Institutional Reviewer Board**: N/A
- **Informed Consent**: N/A
- **Registry and registration no. of the study**: Prospective Register of Systematic Reviews (PROSPERO) (ID: CRD42022369842).
- **Animal studies**: N/A

## Supporting information




**Table S1** Preferred Reporting Items for Systematic Reviews and Meta‐Analyses (PRISMA) checklist.
**Table S2** Detailed search strategy for systematic review.
**Table S3** Newcastle Ottawa Scale (NOS) assessment for Cohort studies.
**Table S4** Pooled analysis of 1‐year subgroup clinical success, Gastroparesis Cardinal Symptom Index (GCSI) score, Gastric Emptying Study (GES) parameters, and adverse events subgrouped by severity.
**Table S5** Factors predicting Gastric Peroral Endoscopic Myotomy (G‐POEM) success or failure.
**Figure S1** Flow diagram of study selection according to the Preferred Reporting Items for Systematic Reviews and Meta‐analyses.
**Figure S2** Funnel plot for 1 year‐clinical success.
**Figure S3** Funnel plot for overall adverse events rate.
**Figure S4** Leave‐One‐Out (LOO) sensitivity analyses for pooled clinical success 1 year after Gastric Peroral Endoscopic Myotomy (G‐POEM).
