# Supplementary material for: Long‐term efficacy (at and beyond 1 year) of gastric peroral endoscopic myotomy for refractory gastroparesis: A systematic review and meta‐analysis
Source: DEN Open. 2024 Oct 4;5(1):e70021. doi: 10.1002/deo2.70021 (PMC11452608; doi:10.1002/deo2.70021)

**SUPPLEMENTARY MATERIALS**

**Supplementary Table 1** Preferred Reporting Items for Systematic Reviews and Meta-Analyses (PRISMA) checklist

| **Section/topic** | **#** | **Checklist item** | **Reported on page #** |
| --- | --- | --- | --- |
| **TITLE** | | |  |
| Title | 1 | Identify the report as a systematic review, meta-analysis, or both. | 1 |
| **ABSTRACT** | | |  |
| Structured summary | 2 | Provide a structured summary including, as applicable: background; objectives; data sources; study eligibility criteria, participants, and interventions; study appraisal and synthesis methods; results; limitations; conclusions and implications of key findings; systematic review registration number. | 3 |
| **INTRODUCTION** | | |  |
| Rationale | 3 | Describe the rationale for the review in the context of what is already known. | 4 |
| Objectives | 4 | Provide an explicit statement of questions being addressed with reference to participants, interventions, comparisons, outcomes, and study design (PICOS). | 4-6 |
| **METHODS** | | |  |
| Protocol and registration | 5 | Indicate if a review protocol exists, if and where it can be accessed (e.g., Web address), and, if available, provide registration information including registration number. | 4 |
| Eligibility criteria | 6 | Specify study characteristics (e.g., PICOS, length of follow-up) and report characteristics (e.g., years considered, language, publication status) used as criteria for eligibility, giving rationale. | 5 |
| Information sources | 7 | Describe all information sources (e.g., databases with dates of coverage, contact with study authors to identify additional studies) in the search and date last searched. | 5 |
| Search | 8 | Present full electronic search strategy for at least one database, including any limits used, such that it could be repeated. | 5 Supplementary table 2 |
| Study selection | 9 | State the process for selecting studies (i.e., screening, eligibility, included in systematic review, and, if applicable, included in the meta-analysis). | 5 Supplementary figure 1 |
| Data collection process | 10 | Describe method of data extraction from reports (e.g., piloted forms, independently, in duplicate) and any processes for obtaining and confirming data from investigators. | 5 |
| Data items | 11 | List and define all variables for which data were sought (e.g., PICOS, funding sources) and any assumptions and simplifications made. | 5,6 |
| Risk of bias in individual studies | 12 | Describe methods used for assessing risk of bias of individual studies (including specification of whether this was done at the study or outcome level), and how this information is to be used in any data synthesis. | 5 |
| Summary measures | 13 | State the principal summary measures (e.g., risk ratio, difference in means). | 5,6 |
| Synthesis of results | 14 | Describe the methods of handling data and combining results of studies, if done, including measures of consistency (e.g., I^2^) for each meta-analysis. | 5,6 |
| Risk of bias across studies | 15 | Specify any assessment of risk of bias that may affect the cumulative evidence (e.g., publication bias, selective reporting within studies). | 5 |
| Additional analyses | 16 | Describe methods of additional analyses (e.g., sensitivity or subgroup analyses, meta-regression), if done, indicating which were pre-specified. | 5,6 |
| **RESULTS** | | |  |
| Study selection | 17 | Give numbers of studies screened, assessed for eligibility, and included in the review, with reasons for exclusions at each stage, ideally with a flow diagram. | 7 |
| Study characteristics | 18 | For each study, present characteristics for which data were extracted (e.g., study size, PICOS, follow-up period) and provide the citations. | 7,  Table 1-5 |
| Risk of bias within studies | 19 | Present data on risk of bias of each study and, if available, any outcome level assessment (see item 12). | 7, Supplementary Figures 2-5 |
| Results of individual studies | 20 | For all outcomes considered (benefits or harms), present, for each study: (a) simple summary data for each intervention group (b) effect estimates and confidence intervals, ideally with a forest plot. | 7-9, Figures 1-3, Supplementary Figures 6-22 |
| Synthesis of results | 21 | Present results of each meta-analysis done, including confidence intervals and measures of consistency. | 7-9, Figures 1-3, Supplementary Figures 6-22 |
| Risk of bias across studies | 22 | Present results of any assessment of risk of bias across studies (see Item 15). | 7, Supplementary Figures 2-5 |
| Additional analysis | 23 | Give results of additional analyses, if done (e.g., sensitivity or subgroup analyses, meta-regression [see Item 16]). | 7-9, Supplementary Figures 6-22 |
| **DISCUSSION** | | |  |
| Summary of evidence | 24 | Summarize the main findings including the strength of evidence for each main outcome; consider their relevance to key groups (e.g., healthcare providers, users, and policy makers). | 9-11 |
| Limitations | 25 | Discuss limitations at study and outcome level (e.g., risk of bias), and at review-level (e.g., incomplete retrieval of identified research, reporting bias). | 9-11 |
| Conclusions | 26 | Provide a general interpretation of the results in the context of other evidence, and implications for future research. | 11 |
| **FUNDING** | | |  |
| Funding | 27 | Describe sources of funding for the systematic review and other support (e.g., supply of data); role of funders for the systematic review. | 11 |

*From:*  Moher D, Liberati A, Tetzlaff J, Altman DG, The PRISMA Group (2009). Preferred Reporting Items for Systematic Reviews and Meta-Analyses: The PRISMA Statement. PLoS Med 6(7): e1000097. doi:10.1371/journal.pmed1000097

**Supplementary Table 2** Detailed search strategy for systematic review

| **Name of database** | **Time span** | **Search strategy** |
| --- | --- | --- |
|  |  |  |
| **PubMed/MEDLINE** | **2012- March 2023** | ("Gastroparesis/therapy"[Mesh]) AND (myotomy OR endoscopic treatment OR endoscopy OR treatment OR peroral myotomy OR endoscopic peroral myotomy OR gastric peroral myotomy OR gastric myotomy) |
| **EMBASE**  **Web of Science**  **Cochrane** | **2012- March 2023**  **2012- March 2023**  **2012- March 2023** | (gastropareses OR 'gastric stasis'/exp OR 'gastric stasis' OR (gastric AND stasis) OR 'gastric stases' OR (gastric AND stases) OR 'stases, gastric' OR (stases, AND gastric) OR 'stasis, gastric' OR (stasis, AND gastric)) AND ('myotomy'/exp OR myotomy OR 'endoscopic treatment' OR (endoscopic AND treatment) OR 'endoscopy'/exp OR endoscopy OR treatment OR 'peroral myotomy' OR (peroral AND ('myotomy'/exp OR myotomy)) OR 'endoscopic peroral myotomy' OR (endoscopic AND peroral AND ('myotomy'/exp OR myotomy)) OR 'gastric peroral myotomy' OR (gastric AND peroral AND ('myotomy'/exp OR myotomy)) OR 'gastric myotomy' OR (gastric AND ('myotomy'/exp OR myotomy)))  (gastropareses OR 'gastric stasis'/exp OR 'gastric stasis' OR (gastric AND stasis) OR 'gastric stases' OR (gastric AND stases) OR 'stases, gastric' OR (stases, AND gastric) OR 'stasis, gastric' OR (stasis, AND gastric)) AND ('myotomy'/exp OR myotomy OR 'endoscopic treatment' OR (endoscopic AND treatment) OR 'endoscopy'/exp OR endoscopy OR treatment OR 'peroral myotomy' OR (peroral AND ('myotomy'/exp OR myotomy)) OR 'endoscopic peroral myotomy' OR (endoscopic AND peroral AND ('myotomy'/exp OR myotomy)) OR 'gastric peroral myotomy' OR (gastric AND peroral AND ('myotomy'/exp OR myotomy)) OR 'gastric myotomy' OR (gastric AND ('myotomy'/exp OR myotomy)))  GPOEM OR G-POEM OR Gastric Peroral Endoscopic Myotomy |

**Supplementary Table 3** Newcastle Ottawa Scale (NOS) assessment for Cohort studies. Tresholds for converting the NOS to AHRQ standards (good, fair, poor):

**Good quality**: 3 or 4 stars in selection domain AND 1 or 2 stars in comparability domain AND 2 or 3 stars in outcome/exposure domain

**Fair quality**: 2 stars in selection domain AND 1 or 2 stars in comparability domain AND 2 or 3 stars in outcome/exposure domain

**Poor quality**: 0 or 1 star in selection domain OR 0 stars in comparability domain OR 0 or 1 stars in outcome/exposure domain

| Studies | Selection | | | | Comparability* | Outcome | | | Total stars |
| --- | --- | --- | --- | --- | --- | --- | --- | --- | --- |
|  |  |  |  |  |  |  |  |  |  |
|  | 1.Representativeness of the exposed cohort | 2. Selection of the unexposed cohort | 3.Ascertainment of exposure | 4.Demonstration that outcome of interest was not present at start of study | 5. Comparability of cohorts on the basis of the design or analysis | 6. Assessment of outcome | 7. Was follow-up long enough for outcomes to occur | 8. Adequacy of follow up of cohorts |  |
|  |  |  |  |  |  |  |  |  |  |
| **Abdelfatah et al. 2021** | ☆ | ☆ | _ | ☆ | ☆ | ☆ | ☆ | ☆ | 7 |
| **Hernandez-Mondragon et al. 2022** | ☆ | ☆ | ☆ | ☆ | ☆ | ☆ | ☆ | ☆ | 8 |
| **Hustak et al. 2020** | ☆ | _ | ☆ | ☆ | _ | ☆ | _ | ☆ | 5 |
| **Labonde et al. 2022** | ☆ | ☆ | ☆ | _ | ☆ | ☆ | ☆ | ☆ | 7 |
| **Tan et al. 2021** | ☆ | ☆ | _ | ☆ | ☆ | _ | ☆ | ☆ | 6 |
| **Ragi et al. 2021** | ☆ | _ | ☆ | ☆ | ☆ | ☆ | _ | ☆ | 6 |
| **Xu et al. 2018** | ☆ | _ | ☆ | _ | ☆ | ☆ | _ | ☆ | 5 |
| **Gregor et al. 2021** | ☆ | _ | _ | ☆ | ☆ | ☆ | _ | ☆ | 5 |
| **Vosoughi et al. 2020** | ☆ | _ | ☆ | _ | ☆ | ☆ | _ | ☆ | 5 |
| **Vosoughi et al. 2022** | ☆ | ☆ | _ | _ | ☆ | ☆ | ☆ | ☆ | 6 |
| **Kahaleh et al. 2018** | ☆ | _ | ☆ | ☆ | _ | _ | ☆ | _ | 4 |
| **Reja et al. 2022** | ☆ | _ | _ | ☆ | ☆ | ☆ | _ | ☆ | 5 |
| **Conchillo 2021** | ☆ | _ | ☆ | _ | ☆ | ☆ | _ | ☆ | 5 |

* A study can be awarded a maximum of one star for each numbered item within the Selection and Exposure categories; a maximum of two stars can be given for Comparability.

**Supplementary Table 4** Pooled analysis of 1-year subgroup clinical success, Gastroparesis Cardinal Symptom Index (GCSI) score, Gastric Emptying Study (GES) parameters, and adverse events subgrouped by severity

| **Outcome** | **Pooled risk** | | | **Heterogeneity** | |
| --- | --- | --- | --- | --- | --- |
|  | **Events/total** | **Estimate** | **95% CI** | **I^2^** | **Cochrane's Q test, p-value** |
| Pooled 1-year clinical success, for studies including patients with refractory gastroparesis and 4 h PGR > 10% | 137/228 | 0.610 | 0.526, 0.709 | 46.66% | Q= 5.972, p=0.131 |
| Pooled 1-year clinical success, for studies defining CS as a decrease of 1 point in the total GCSI, with a reduction of at least 25% in two GCSI subscales | 435/526 | 0.677 | 0.472, 0.970 | 95.85% | Q= 153.833, p<0.001 |
| Pooled mean pre-procedural GCSI score | - | 3.369 | 3.095, 3.644 | 98.29% | Q= 585.757, p<0.001 |
| Pooled mean GCSI score 1 year after G-POEM | - | 1.746 | 1.369, 2.122 | 97.99% | Q= 447.039, p<0.001 |
| Pooled mean GCSI score 2 years after G-POEM | - | 1.339 | 0.857, 1.820 | 96.74% | Q= 91.955, p<0.001 |
| Pooled mean difference between the 1-year-post-procedure and pre-operative GCSI scores | - | -1.615 | -2.185, -1.045 | 98.65% | Q= 668.866, p<0.001 |
| Pooled mean difference between the 2-year-post-procedure and pre-operative GCSI scores | - | -2.187 | -2.250, -2.124 | 0% | Q= 1.527, p=0.676 |
| Pooled mean pre-procedural gastric t ½ (minutes) | - | 234.578 | 207.962, 261.195 | 92.95% | Q= 56.775, p<0.001 |
| Pooled mean pre-procedural 4 h PGR | - | 42.585 | 37.897, 47.273 | 72.62% | Q= 14.610, p=0.006 |
| Pooled mean post-procedural gastric t ½ (minutes) | - | 127.833 | 47.604, 208.062 | 99.6% | Q= 755.927, p<0.001 |
| Pooled mean post-procedural 4 h PGR | - | 15.666 | 9.976, 21.356 | 76% | Q= 12.498, p=0.006 |
| Pooled mean difference between post-operative and pre-operative gastric t ½ (minutes) | - | -106.824 | -183.285, -30.363 | 98.87% | Q= 353.872, p<0.001 |
| Pooled mean difference between post-operative and pre-operative 4 h PGR | - | -27.326 | -34.556, -20.096 | 68.14% | Q= 9.416, p=0.024 |
| Pooled rate of mild AEs, classified according to the ASGE lexicon classification | 64/906 | 0.069 | 0.054, 0.086 | 0% | Q= 10.517, p=0.485 |
| Pooled rate of moderate AEs, classified according to the ASGE lexicon classification | 12/906 | 0.015 | 0.008, 0.024 | 0% | Q= 8.202, p=0.695 |
| Pooled rate of severe AEs, classified according to the ASGE lexicon classification | 1/906 | 0.006 | 0.002, 0.011 | 0% | Q= 5.612, p=0.898 |

CI Confidence Intervals, PGR Percent Gastric Retention, GCSI Gastroparesis Cardinal Symptom Index, CS clinical success, t ½ half emptying time, AE adverse event, ASGE American Society for Gastrointestinal Endoscopy

**Supplementary Table 5** Factors predicting G-POEM success or failure

| **Author** | **Factors predictive**    **Success Failure** | |
| --- | --- | --- |
| Abdelfatah et al. | - | Higher BMI and psychiatric medication use, history of pain medication, longer duration of GP (multivariate analysis) |
| Hernandez Mondragon et al. | Diabetic etiology, early diagnosis of <24 months, predominant nausea and vomiting, GCSI score between 1.5 and 2.5 at 6 months, and 4 h PGR <10% at 6 months predictive of long-term clinical success (multivariate analysis) | - |
| Labonde et al. | High preoperative GCSI bloating subscale (univariate) | High preoperative nausea subscale score (univariate) |
| Vosoughi et al. | Post G-POEM CSA at 40-mL (>154 mm^2^) and 50-mL (>247.5 mm^2^) distention volume (multivariate analysis) | - |
| Ragi et al. | High pre-operative GCSI satiety subscale (multivariate analysis) | High 4 h PGR (multivariate analysis) |
| Vosoughi et al. | GCSI > 2.6, 4 h PGR > 20% (multivariate analysis) clinical success at 1 months (univariate analysis) predictors of clinical success at 12 months |  |
| Xu et al. | Age, GCSI (< 30) and GES (2 h PGR > 78% and t ½ <221.6 minutes) associated with favourable clinical outcomes (univariate) | - |
| Conchillo et al. | Pyloric DI improvement associated with clinical success at 6 months (univariate analysis) | - |

GP gastroparesis, BMI Body Mass Index, PGR Percent Gastric Retention, DI distensibility index, GCSI Gastric Cardinal Symptom Index, GES Gastric Emptying Study, CSA Cross Sectional Area

**Supplementary Figure 1** Flow diagram of study selection according to the Preferred Reporting Items for Systematic Reviews and Meta-analyses


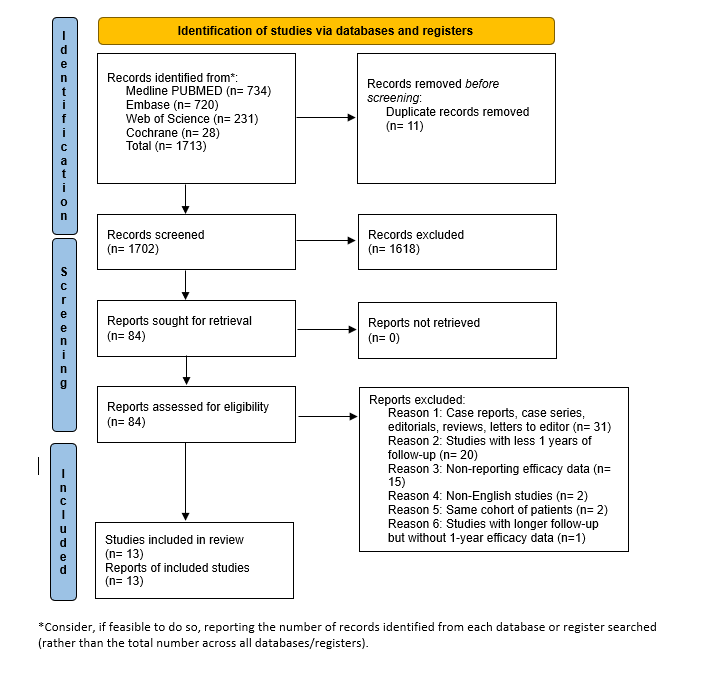


**Supplementary Figure 2** Funnel plot for 1 year-clinical success


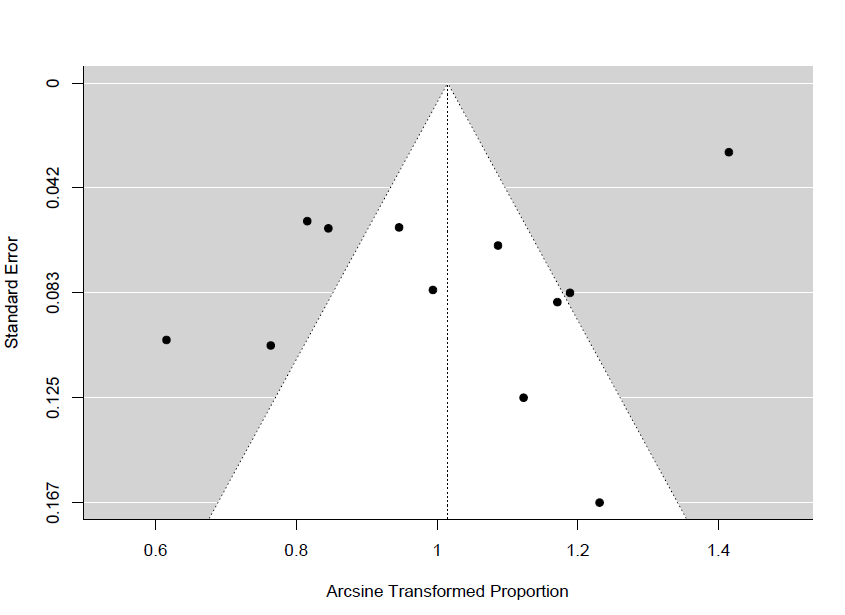


**Supplementary Figure 3** Funnel plot for overall adverse events rate


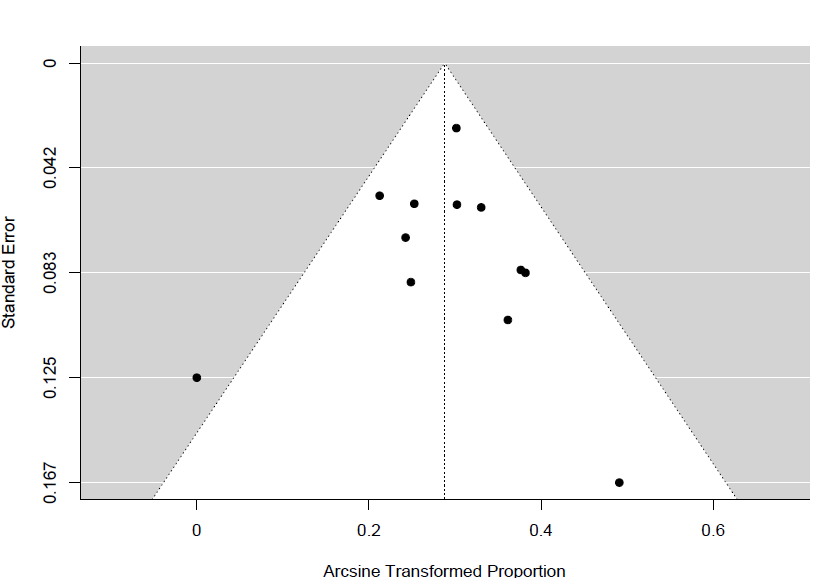


**Supplementary Figure 4** Leave (LOO) sensitivity analyses for pooled clinical success rate 1 year after G-POEM


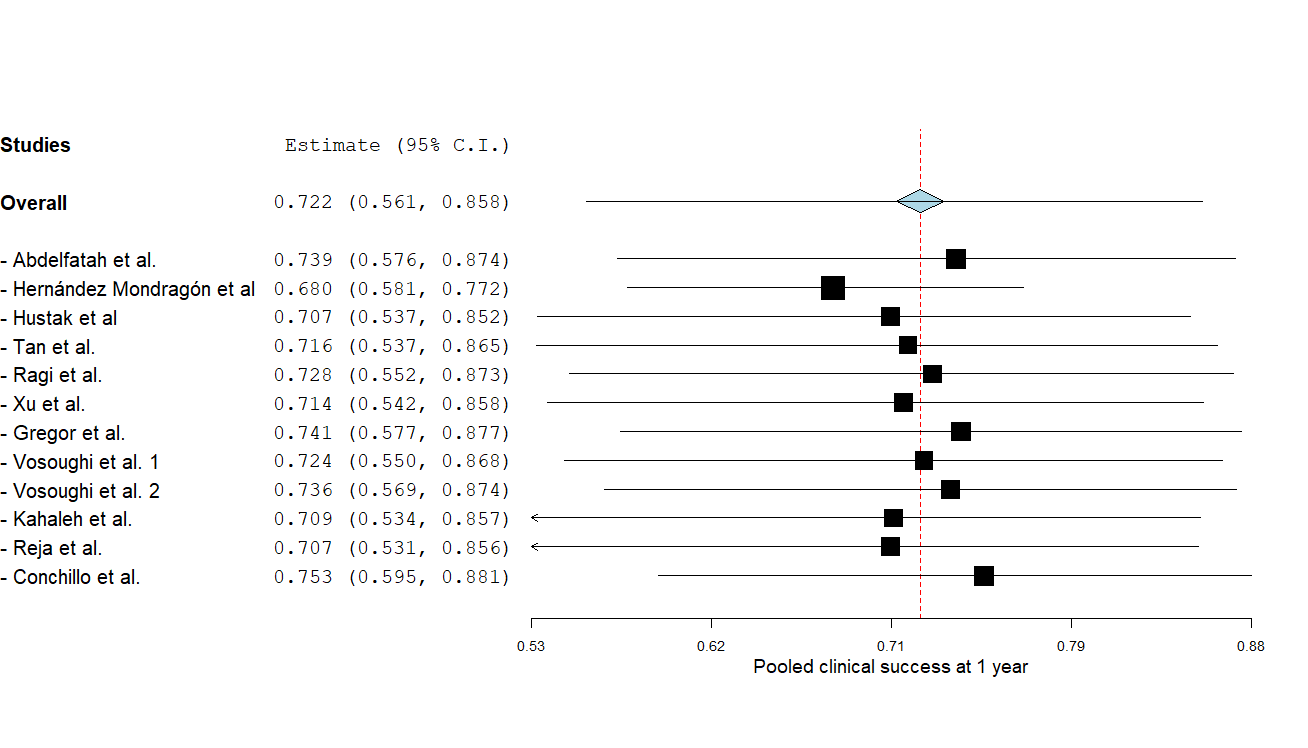

Supplement: Supplementary file 1 — Table S1 Preferred Reporting Items for Systematic Reviews and Meta‐Analyses (PRISMA) checklist. Table S2 Detailed search strategy for systematic review. Table S3 Newcastle Ottawa Scale (NOS) assessment for Cohort studies. Table S4 Pooled analysis of 1‐year subgroup clinical success, Gastroparesis Cardinal Symptom Index (GCSI) score, Gastric Emptying Study (GES) parameters, and adverse events subgrouped by severity. Table S5 Factors predicting Gastric Peroral Endoscopic Myotomy (G‐POEM) success or failure. Figure S1 Flow diagram of study selection according to the Preferred Reporting Items for Systematic Reviews and Meta‐analyses. Figure S2 Funnel plot for 1 year‐clinical success. Figure S3 Funnel plot for overall adverse events rate. Figure S4 Leave‐One‐Out (LOO) sensitivity analyses for pooled clinical success 1 year after Gastric Peroral Endoscopic Myotomy (G‐POEM). [file DEO2-5-e70021-s001.docx]
